# *Blastocystis* spp. and Other Intestinal Parasites in Polish Soldiers Deployed to Lebanon and Iraq

**DOI:** 10.3390/pathogens13030271

**Published:** 2024-03-21

**Authors:** Danuta Izabela Kosik-Bogacka, Krzysztof Korzeniewski, Natalia Łanocha-Arendarczyk, Joanna Korycińska, Małgorzata Lepczyńska, Ewa Dzika, Małgorzata Marchelek-Myśliwiec

**Affiliations:** 1 Independent Laboratory of Pharmaceutical Botany, Pomeranian Medical University in Szczecin, 70-111 Szczecin, Poland; 2 Department of Epidemiology and Tropical Medicine, Military Institute of Medicine—National Research Institute, 04-141 Warsaw, Poland; kkorzeniewski@wim.mil.pl; 3 Department of Biology and Medical Parasitology, Pomeranian Medical University in Szczecin, 70-111 Szczecin, Poland; natalia.lanocha.arendarczyk@pum.edu.pl; 4 Department of Medical Biology, School of Public Health, University of Warmia and Mazury, 10-561 Olsztyn, Poland; joanna.korycinska@uwm.edu.pl (J.K.); mlepczynska@gmail.com (M.L.); e.dzika@uwm.edu.pl (E.D.); 5 Clinic of Nephrology, Transplantology and Internal Medicine, Pomeranian Medical University in Szczecin, 70-111 Szczecin, Poland; malgorzata.marchelek@gmail.com

**Keywords:** *Blastocystis* spp., intestinal protozoa, military personnel

## Abstract

Intestinal parasitic infections are one of the most common infectious diseases worldwide, particularly in developing countries. A distinct group at increased risk of infection is military personnel deployed overseas for extended periods, typically six months at a time. The aim of this study was to determine the prevalence of *Blastocystis* spp. and other intestinal parasites in Polish military personnel returning from deployments to Lebanon (n = 206) and Iraq (n = 220). In this group of subjects, we found *Blastocystis* spp. (13.6%), *Dientamoeba fragilis* (3.3%), *Entamoeba coli* (0.9%), and *Endolimax nana* (0.5%). *Entamoeba histolytica* sensu lato and *Chilomastix mesnili* infections were detected only in one soldier returning from Lebanon and Iraq, respectively. *Blastocystis* subtype (ST) 3 was predominant in soldiers returning from Lebanon, followed by ST2 and ST1. ST1 infection was predominant in soldiers returning from Iraq, followed by ST3 and ST2. Our study affirms that, deployment abroad is of no influence of the prevalence of parasitic protozoa. However, it would be worth to monitor parasite infection in military personnel returning from tropical zone even if they have no actual symptoms. In addition, it is very important to determine the subtypes of *Blastocystis*—this may help to clearly define their pathogenicity, especially considering the scarcity of studies on *Blastocystis* genotypes in Iraqi and Lebanese residents.

## 1. Introduction

Intestinal parasite infection is a prevalent global infectious disease, particularly impacting developing countries. Military personnel, deployed overseas for extended periods, face an elevated risk of infection, with studies establishing a direct correlation between their duration of stay in a foreign country and increased susceptibility to travel-related illnesses [1].

The major causes of gastrointestinal symptoms, including diarrhea, abdominal pain, nausea, vomiting, bloating, and weight loss, are intestinal protozoal infections such as *Giardia duodenalis*, *Cryptosporidium* spp., and *Cyclospora cayetanensis*. In addition, while *Entamoeba histolytica* sensu stricto is rarely found, its early diagnosis is crucial due to the potentially invasive nature of this parasite [2,3]. In comparison, intestinal worm infections generally do not lead to serious clinical complications, except for *Strongyloides stercoralis* [4]. Given this, laboratory diagnosis of intestinal protozoal and helminth infections in returning military personnel holds significant importance.

Returning travelers commonly report *Blastocystis* spp. infection [5,6]. *Blastocystis* spp., prevalent in various animals globally, exhibits human infection rates ranging from 0.5% to 30% in developed countries and from 30% to 76% in developing countries [7]. Transmission occurs through various routes, including fecal-oral transmission and ingestion of contaminated water and food [8,9]. The pathogenicity of *Blastocystis* spp. remains a matter of debate. Available research suggests it can be classified as a parasite and its pathogenicity depends on factors such as interaction with the gut microbiota, subtype, and the host immune response [10,11,12]. *Blastocystis* spp. comprises 34 subtypes (ST1-ST34), of which 14 have been reported in humans (ST1-10, ST12, ST14, ST16, and ST23) with ST1-ST4 causing the most frequent infections [13]. 

Gastroenteritis is a significant concern in military deployments associated with *Blastocystis* spp. infection and concurrent presence of *Dientamoeba fragilis* [14,15,16,17]. *D. fragilis*, recognized as a potentially pathogenic gastrointestinal organism, globally ranges in prevalence from <1% to 80% depending on study parameters [17,18]. However, the protozoan’s role in causing chronic gastrointestinal symptoms is increasingly being questioned [19].

The prevalence of intestinal protozoan parasites and the distribution of *Blastocystis* subtypes in military personnel remain understudied. Molecular techniques reveal geographic variations in *Blastocystis* spp. prevalence and distribution worldwide, with certain regions such as the Middle East remaining understudied [20,21]. In the available scientific literature, there is a lack of data concerning the subtypes of *Blastocystis* reported in Iraq and Lebanon. Therefore, the objectives of this study are to ascertain the prevalence of *Blastocystis* spp. and other intestinal parasites in Polish military personnel returning from deployments to Lebanon and Iraq.

## 2. Materials and Methods

### 2.1. Ethics Statement

The study was conducted in accordance with the tenets of the Declaration of Helsinki, and the research protocol was approved by the Bioethics Committee of the Pomeranian Medical University in Szczecin and the EU guidelines on good clinical practice for medical device trials in the European Community. Patients enrolled in the study were informed about the study procedure and signed an informed consent form. 

### 2.2. Study Population 

Participants were Polish military personnel stationed for 6 months in southern Lebanon near the border with Israel (n = 206) and in various parts of Iraq (n = 220), who returned in February 2023 and April 2023, respectively. This was a repeat deployment for 80% of the soldiers. Parasitologic examinations of stool samples were performed between 1 and 3 weeks after return. The soldiers, 92% of whom were men and 8% of whom were women, came from different parts of Poland and were divided into three age groups (Table 1). Exclusion criteria included the use of antibiotics and drugs for parasitic infections or having undergone radiological examinations with contrast agents.

Before and after departure, the health status of all soldiers was validated by a military medical board. In accordance with prevailing regulations, optimal general health served as a prerequisite for engaging in military service abroad. No antiparasitic drugs were administered to military personnel either before their departure or during their stay in Iraq and Lebanon.

Demographic information, encompassing age, gender, and place of residence, along with details about current and past missions, mission durations, and epidemiological conditions in the mission area, including water supply characteristics, consumption of local food, interaction with the local population and animals, and the presence of gastrointestinal symptoms, were systematically collected through questionnaires from individuals engaged in military service abroad. Officers, noncommissioned officers, and enlisted personnel who interacted with the local population, consumed fruits and vegetables from the soldiers’ mess at the military base, drank bottled water and used chlorinated water for bathing in the sanitary facilities, and denied contact with animals were included in the parasitological study.

### 2.3. Study Area

Soldiers in Lebanon were stationed in the southern provinces of Nabatieh and Lebanon-Sud. Lebanon is a country located in western Asia in the Middle East, bordering Syria and Israel on the Mediterranean Sea. The country has a subtropical Mediterranean climate, with average winter and summer coastal temperatures of ~10 °C and 30 °C, respectively. In mountainous areas above 1000 m above sea level, the temperature is 6 °C and 22 °C, respectively. The dry season lasts from June to September [22]. In Iraq, the main component of the troops was stationed at Al-Asad Air Base (Al-Anbar Governorate), northwest of Baghdad. Iraq is a country in Southwest Asia, in the central part of the Middle East, on the Persian Gulf, bordering Iran, Jordan, Kuwait, Saudi Arabia, Syria, and Türkiye. Iraq has a tropical climate zone and a subtropical zone in the north. The temperature in Iraq varies, during the summer season it exceeds 40 °C during the day, at night in the deserts it can drop to 5 °C. In winter, average daytime temperatures are around 10–20 °C, with frosts common at night. The mountainous edges of Iraq are cool, with summer temperatures below 20 °C [23].

### 2.4. Stool Examination

Prior to and after departure, all soldiers underwent a stool parasitological examination (three times with an interval of 2–3 days). 

Participants received stool sample containers and standard instructions for proper and safe collection and preservation of the samples. Urine-free stool samples were collected in a clean, dry, leak-proof container. Stool samples from all patients were examined macroscopically for consistency (i.e., watery, loose, or firm), presence of mucus, and adult worms (tapeworms proglottids, roundworms). 

All stool samples from the soldiers were examined by direct preparation microscopy with normal saline (0.9%) and Lugol’s solution, sedimentation with distilled water, and Fülleborn flotation [24,25]. The slides were examined under both low-power (10×) and high-dry (40×) magnification under a light microscope. A detailed description of the methods used can be found in our previous work [26].

The intensity of parasite infection was determined at a magnification of ×40 using four levels of parasite load: (+) low—single protozoa in almost every field of view, (++) medium—5 to 10 protozoa in every field of view, (+++) high—>10–20 protozoa in every field of view. Only the stool samples positive for *Blastocystis* spp. were subjected to conventional PCR (cPCR).

### 2.5. Molecular Assays for Blastocystis Identification

Positive stool samples stored in 70% ethanol were used for DNA extraction and molecular analysis. From each sample, 200 mg of stool was washed three times in phosphate-buffered saline (PBS) using 2.0 mL microcentrifuge tubes (centrifugation at 2700× *g* for 5 min). Total DNA was extracted according to the manufacturer’s protocol Qiamp DNA Stool Mini Kit (Qiagen, Valencia, CA, USA). The extracted DNA was eluted in 40 μL TE buffer and stored at −20 °C until further analysis.

The SSU rDNA gene was amplified using primers RD5 (5′-ATCTGGTTGATCCTGCCAGT-3′) and BhRDr (3′-GAGCTTTTTAACTGCAACAACG-5′). These primers amplify a ~607 base pair (bp) fragment of the 1.8 kbp small subunit ribosomal RNA (SSU rRNA) gene [27]. The PCR reaction was performed in a final volume of 25 µL. The amplification mixture contained 12.5 µL of 2 × PCR Master Mix Plus (0.1 U/µL Taq polymerase supplied in a PCR buffer, 4 mM MgCl_2_, and 0.5 mM of each dNTP) (A&A Biotechnology, Gdynia, Poland), 0.1 μM of each primer, 1.5–2 μL of genomic DNA and distilled water. Amplification was performed under the following conditions: initial denaturation at 94 °C for 4 min, followed by 30 cycles of denaturation at 95 °C for 15 s, annealing at 60 °C for 15 s, extension at 72 °C for 30 s, and final extension at 72 °C for 5 min [27]. In addition, to determine *Blastocystis* subtypes, STS-PCR was performed using primers specific for five subtypes (ST1-ST4 and ST7) previously described by Yoshikawa et al. [28].

Cycling conditions consisted of an initial denaturation step at 94 °C for 5 min, followed by 35 cycles at 94 °C for 30 s, a variable annealing temperature depending on the primer combination (ST1-56 °C, ST2-66 °C, ST3-61 °C, ST4-50 °C) for 30 s, and an extension step at 72 °C for 40 s, followed by a final extension step at 72 °C for 5 min [29]. For ST7, cycling conditions consisted of an initial denaturation step at 94 °C for 5 min, followed by 40 cycles at 94 °C for 30 s, an annealing temperature of 57 °C for 30 s, and an extension step at 72 °C for 1 min, followed by a final extension step at 72 °C for 5 min [30]. PCR amplification reactions were performed in a Mastercycler Nexus (Eppendorf, Hamburg, Germany). 

Products were visualized by electrophoresis in 1.5% agarose gels with Midori Green Advance (Genetics) using GelDocXR (Bio-Rad, Hercules, CA, USA). Amplicons were purified using the QIAquick PCR Purification Kit and sequenced in both directions by Macrogen Humanizing Genomics Europe (Amsterdam, The Netherlands). Consensus sequences were aligned using BioEdit Sequence Alignment Editor v. 7.1.10 and analyzed using BLAST (www.ncbi.nlm.nih.gov/BLAST, accessed on 18 December 2023). 

### 2.6. Statistical Methods

All calculations were performed using the StatSoft Inc. statistical package. STATISTICA (data analysis software system) version 10.0. (www.statsoft.com, accessed on 4 September 2023). The qualitative variables were presented with the use of count and percentage. 

## 3. Results

Before departure, no developmental forms of parasites were detected in the stool samples of military personnel. Upon returning from peacekeeping missions, only intestinal protozoa were observed in 17.1% (n = 73) of soldiers, with 20.4% (n = 42) among those returning from Lebanon and 13.7% (n = 31) among those returning from Iraq. In the parasitological examination of the feces, adult tapeworms, proglottids, and roundworms were not identified. Diarrhea was found in 32 soldiers, including 10 returning from Iraq and 22 from Lebanon. Two soldiers returning from Lebanon with diarrhea were found to be infected with *Blastocystis* spp.

**Figure 1:**
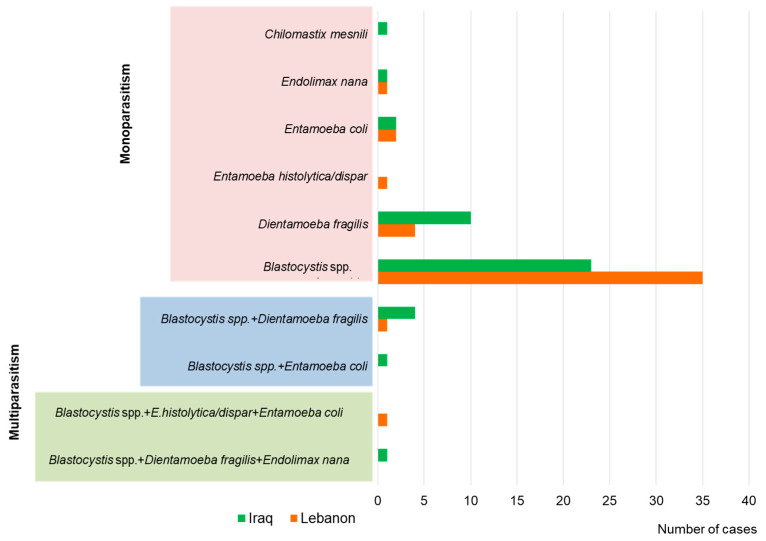
Species distribution of intestinal parasites detected among the Polish soldiers stationed in Iraq and Lebanon (all cases of parasitosis were detected by general stool parasitologic examination and, if *Blastocystis* spp. was detected, additional molecular PCR testing was performed).

Among returnees from Lebanon, *Blastocystis* spp. was most commonly detected (17%), and the intensity of infection was usually low (Table 2). *D. fragilis* was detected in 1.9% of participants, and the intensity of infection was low and moderate. Co-infection with *Blastocystis* spp. and *D. fragilis* was found in one soldier, and the intensity of infection was moderate (Figure 1). Co-infection with *E. histolytica* sensu lato (low) and *E. coli* (medium) was found in one participant with high forms of *Blastocystis* spp. In addition, non-pathogenic gastrointestinal endobionts were found in two soldiers, including single *E. coli* cysts in one soldier and single *E. nana* cysts in another.

*Blastocystis* spp. was also the most common organism found in soldiers returning from Iraq (10.5%), and the infection intensity was usually high (Table 2). *D. fragilis* was found in 4.5% of participants, and the intensity of infection was mostly moderate and low. Co-infection with *Blastocystis* spp. and *D. fragilis* was found in 1.8%. High-intensity *Blastocystis* spp. and medium-intensity *D. fragilis* infections were found in three patients. Medium-intensity infection was found in one patient. One patient had co-infection with *Blastocystis* spp. (medium intensity) and *E. coli* (low intensity). In addition, single cysts of *E. coli* were found in other soldiers, and single cysts of non-pathogenic *Chilomastix mesnili* were found in another.

Genotyping results showed the presence of three different STs in 53 of 58 isolates (91.4%), while five samples remained undefined (8.6%) (Table 3). ST3 (53.3%) was most frequently found in soldiers returning from Lebanon, followed by ST2 (26.7%) and ST1 (20.0%). ST1 (52.2%) infection was predominant in soldiers returning from Iraq, followed by ST3 (39.1%) and ST2 (8.7%). For co-infection with *Blastocystis* spp. and *D. fragilis*, the ST1 was found in one participant returning from Lebanon and ST3 or ST1 in soldiers returning from Iraq. ST1 and ST3 were found in two soldiers with diarrhea.

## 4. Discussion

### 4.1. Prevalence of Blastocystis spp.

Data on the prevalence of *Blastocystis* spp. in the Middle East, a region where foreign military personnel have been stationed for years, are incomplete and variable [21]. Among others, in the local population, the prevalence of *Blastocystis* spp. has been determined in Iran (<1%) and Lebanon (<20%), but in Saudi Arabia and Syria the prevalence is higher, reaching almost 70% [31,32]. In Iraq, the prevalence of *Blastocystis* spp. ranges from 12% to 33% depending on the age of the subjects, but in patients with gastrointestinal disorders, the range is 17–60% [32,33,34,35,36]. Among Jordanians with diarrhea, the frequency of *Blastocystis* spp. ranged from <1% to 60% [36,37,38]. Residents of the Middle East region are often exposed to the risk of parasitic infections due to precarious sanitary conditions, inadequate drinking water supply, and contact with animals [18,21,39]. Few studies have been conducted in Lebanon, and most of them have included children, with prevalence rates ranging from 20% to 60% [40]. Based on available data from the scientific literature, the prevalence of *Blastocystis* spp. in Poland was found to range from 0.14% to 23.6%, depending on the study group [13].

In this study of military personnel, we found that the prevalence of *Blastocystis* spp. in Lebanon and Iraq did not exceed 17% and ~11%, respectively, and the difference may be due to population size, survey techniques, and parasite exposure factors. In the study on Polish soldiers participating in peacekeeping missions in Afghanistan and Iraq before 2010, *Blastocystis* spp. infection was found in ~15%, although it needs to be stressed that only direct stool examination was used [16]. A similar prevalence was observed in Polish soldiers stationed in the Republic of Kosovo (Southern Europe) [41]. A twofold higher prevalence of *Blastocystis* spp. was found in Thai soldiers [42], although the conditions of deployment and the climatic zone, in Southeast Asia, differed significantly. A much lower prevalence of *Blastocystis* spp. infection (6.2%) was found in active-duty military or military beneficiaries during Operation Iraqi Freedom [15]. A similar 7% prevalence has also been found in US military personnel deployed to the Middle East [43]. 

In our study, we found only ST1, ST2, and ST3 subtypes of *Blastocystis* spp. in Polish soldiers returning from peacekeeping missions in Lebanon and Iraq. ST3 was the dominant subtype in soldiers returning from Lebanon. It is the most human-specific subtype, suggesting anthroponotic transmission [44]. It is also found in a range of symptomatic and asymptomatic individuals. Similarly, ST3 predominated in the study on Syrian refugees living in informal tented settlements in northern Lebanon, followed by either ST1 or ST2 [21]. Similarly, in slaughterhouse workers and hospital patients from Lebanon, the predominant *Blastocystis* spp. subtype was ST3, folowed by ST1 and then ST2 [45]. In contrast, the frequencies of all these three subtypes were similar in patients from six hospitals in northern Lebanon [36].

In the present study, ST1 was the dominant subtype in Polish soldiers returning from Iraq. ST1 is the dominant subtype in symptomatic patients, but no significant association between subtypes and gastrointestinal symptoms has been found [36]. In addition, human ST1 isolates have been associated with increased pathogenicity in experimentally infected rats [46]. ST1 has also been associated with zoonotic transmission from livestock, including studies on the Libyan population [7]. The study by Salehi et al. [47] in Iraq indicates that ST1, ST2, and ST3 are the most commonly recorded subtypes in Iraq, with phylogenetic analysis showing a high probability of zoonotic transmission of *Blastocystis* spp. However, ST3 (25%), ST1 (18.8%), ST5 (11.3%), and ST7 (11.3%) were found in patients with gastrointestinal diseases in Kirkuk province in Iraq [48]. In the cases of co-infection with *Blastocystis* spp. and *D. fragilis*, the ST1 subtype was found in participants returning from Lebanon and ST3 or ST1 in soldiers returning from Iraq.

It is possible that the subtypes of *Blastocystis* spp. found in the soldiers in this report indicate the possibility of infection with this protozoan during deployment, as ST1-ST4, ST6, ST7, and ST9 are most common in the Zachodniopomorskie Voivodship from which the soldiers came, and ST1-ST3, ST6, and ST7 in the Mazowieckie province [13].

Interestingly, ST4 was not found in the human cohorts analyzed in the present study. The absence of this subtype in the human population, or its very low prevalence, has already been described in Lebanon and more generally in Middle Eastern countries [36,40]. To date, little is known about how *Blastocystis* subtypes spread and why ST4 is absent in some parts of the world, including Lebanon, and seems to be common mainly in Europe [40,49]. Forsell et al. [49] noted that in regions of the world where ST1 is predominant, the ST4 subtype is rare. 

It has been suggested that humans can be infected with *Blastocystis* spp. of zoonotic origin without direct contact with animals. Soldiers from Iraq and Lebanon in this study reported no contact with animals, but zoonotic *Blastocystis* spp. can be transmitted to humans by ingestion of *Blastocystis*-contaminated food and water. In Iraq, Khoshnood et al. [50] also noted the presence of ST4 and ST7; these subtypes, such as ST3, produce cysteine proteases that can cleave human IgA in vitro, and this has been suggested as a mechanism for the survival and colonization of *Blastocystis* spp. in the gut [51,52]. The presence of ST4 and ST7 has been reported in soldiers on peacekeeping missions in southern Europe [41]. The authors noted that ST7 is more frequently detected in Asia than in Europe and has a greater avian zoonotic potential. It was suggested that the occurrence of rarer *Blastocystis* spp. subtypes in the Polish soldier population (atypical for a particular geographic region) may be related to the presence of other nationalities comprising the military contingent and the likely risk of anthroponotic transmission. 

*Blastocystis* spp. is transmitted via the fecal-oral route and can occur via contaminated water or food, human-to-human, or animal-to-human contacts [11]. With soldiers stationed in Iraq and Lebanon reporting the possibility of eating local foods, including fruits and vegetables, there is a real risk of transmission of protozoan organisms, including *Blastocystis* spp. or *D. fragilis*, along with contaminated food.

### 4.2. Co-Infection with Blastocystis spp. 

*Blastocystis* spp. and *D. fragilis* are commonly found in both diarrheic and non-diarrheic patients [53]. The global prevalence of *D. fragilis* is estimated to be between 0.4 and 71%, with a higher prevalence in developed countries [17]. In this study, *D. fragilis* infection was detected in 3.3% of soldiers returning from Iraq and Lebanon. A similar infection rate (5%) has been found in U.S. Army soldiers deployed near Alexandria (Egypt), but those soldiers had diarrhea [54]. In contrast, much higher rates of *D. fragilis* infection were found in people living in the area. It ranged from about 40% of workers in a pastry factory in northern Lebanon [55] to 60% of children in schools in Tripoli (Lebanon) [40].

Clinical symptoms associated with *D. fragilis* infection include abdominal pain, loose stools, and diarrhea; in addition, *Blastocystis* spp. may be associated with abdominal pain, diarrhea, constipation, nausea, flatulence, bloating, cramps, nausea, and fatigue [17,44]. In the active duty population during Operation Iraqi Freedom, multiprotozoal coinfections of *Blastocystis* spp. with *Chilomastix mesnili* (n = 1), *E. nana* (n = 3), *E. coli* (n = 4), *E. histolytica* (n = 1), and *E. nana* (n = 1) [15]. In the study presented here, co-infections by *Blastocystis* spp. and *D. fragilis* were found in 5 participants among military personnel from Iraq and Lebanon. Piubelli et al. [56] found a significant association between *Blastocystis* and *D. fragilis*, which may indicate a cooperative interaction between the two protozoa. The role of co-infection is unclear, but it is hypothesized that *D. fragilis* is transmitted via the fecal-oral route and may be associated with travel and animal contact [57]. It has also been noted that co-infection of these two intestinal parasites has a prevalence ranging from ~18 to 34% in patients with gastrointestinal symptoms [58]. In the present study, co-infection of *Blastocystis* spp. with (1) *E. coli*, (2) *D. fragilis*, and *E. nana* and (3) *E. histolytica*/*dispar* and *E. coli* was found in a small number of participants.

This study has at least two notable limitations. First, only direct microscopy was used for the identification of intestinal parasites. Only in the case of *Blastocystis* spp. infection was the stool material subjected to molecular testing. Second, the compositions of the patients’ microbiota were not examined. The main strength of this study is that, to our knowledge, this is the only research on *Blastocystis* subtypes in soldiers returning from deployments to Iraq and Lebanon.

## 5. Conclusions

Our study affirms that deployment abroad is of no influence on the prevalence of parasitic protozoa. However, it would be worth monitoring parasite infection in military personnel returning from tropical zones even if they have no actual symptoms. In addition, it is very important to determine the subtypes of *Blastocystis*—this may help to clearly define their pathogenicity, especially considering the scarcity of studies on *Blastocystis* genotypes in Iraqi and Lebanese residents. 

## Figures and Tables

**Table 1:** Characteristics of military personnel who participated in the study (M, male; F, female; T, total).

Characteristic	Lebanon			Iraq			Total		
	M	F	T	M	F	T	M	F	T
M/F: n (%)	185	21	206	209	11	220	394	32	426
Age (years):									
<35	53	11	64	52	5	57	105	16	121
35–45	98	9	107	108	4	112	206	13	219
>46	34	1	35	49	2	51	83	3	86

**Table 2:** Intensity of *Blastocystis* spp. and *Dientamoeba fragilis* infections in soldiers returning from peacekeeping missions in Iraq and Lebanon.

Species	Prevalence of Infection	Intensity of Infection (n/%)		
		+	++	+++
Lebanon				
*Blastocystis* spp.	17%	17/48.5	11/31.4	7/20.0
*D. fragilis*	1.9%	2/50.0	2/50.0	-
Iraq				
*Blastocystis* spp.	10.5%	5/21.7	8/34.8	10/43.5
*D. fragilis*	4.5%	5/50.0	4/40.0	1/10.0

**Table 3:** Subtype distribution of *Blastocystis* spp. in returning soldiers and co-infection infection.

Soldiers Returning from:	Subtype of *Blastocystis* spp.			
	ST1 (n)	ST2 (n)	ST3 (n)	Unknown (n)
single infection				
Lebanon	5	7	14	5
Iraq	11	2	7	-
co-infection with				
*Blastocystis* spp. and *D. fragilis*				
Lebanon	-	-	1	-
Iraq	1	-	2	-
*Blastocystis* spp. and *D. fragilis* and *E. nana*				
Lebanon	-	-	1	
*Blastocystis* spp. and *Entamoeba histolytica*/*dispar* and *E. coli*				
Lebanon	-	1	-	
*Blastocystis* spp. and *E. coli*				
Lebanon	1	-		

## Data Availability

The sequences of *Blastocystis* spp. Obtained in this study are deposited in GenBank (accession nos.: OR978579; OR978494; OR978406; OR978404; OR978372, PP406900–PP406925; PP396705–PP396718). Other data presented in this study are available upon request from the corresponding author.
